# Polyphenol analysis in human milk by a rapid, cost-effective, and accurate micromethod: translational development

**DOI:** 10.1590/1984-0462/2024/42/2023186

**Published:** 2024-03-25

**Authors:** Mariela Valentina Cortez, Ana Veronica Scotta, Agustín Ramiro Miranda, Elio Andrés Soria

**Affiliations:** a Universidad Nacional de Córdoba, Facultad de Ciencias Médicas, Escuela de Fonoaudiología, Ciudad de Córdoba, Córdoba, Argentina.; b Montpellier Interdisciplinary Center on Sustainable Agri-food Systems (MoISA), University of Montpellier, CIRAD, CIHEAM-IAMM, INRAE, Institut Agro, IRD, Montpellier, France.; c Universidad Nacional de Córdoba, Facultad de Ciencias Médicas, Cátedra de Biología Celular, Histología y Embriología, Instituto de Biología Celular, Ciudad de Córdoba, Córdoba, Argentina.

**Keywords:** Analytic sample preparation methods, Clinical laboratory techniques, Milk, human, Polyphenols, Technology assessment, biomedical, Métodos analíticos de preparação de amostras, Técnicas de laboratório clínico, Leite humano, Polifenóis, Avaliação de tecnologia biomédica

## Abstract

**Objective:**

To develop a rapid method for analysing polyphenols, which are potentially active antioxidants against neonatal oxidative stress, from small human milk (HM) volumes.

**Methods:**

Acid and alkaline extractions were compared using two dyes: Folin-Ciocalteu and Fast Blue BB. Linearity, sensitivity, recovery percentage, polyphenol content, precision, and stability were assessed in 14 HM samples and compared using the Kruskal-Wallis H test (p<0.05). The best technique was applied to 284 HM samples to determine their polyphenolic content and its association with maternal diet by multifactorial linear regression.

**Results:**

Acidic extraction successfully recovered the gallic acid reference standard, whereas alkaline extraction overestimated it. Calibration curves for all methods were linear (R^2^>0.96) up to 500 mg/L. All bicarbonate-based Folin-Ciocalteu methods assayed were stable and repeatable, whereas Fast Blue BB-based variants were not. HM polyphenols (mean=94.68 mg/L) positively correlated to the dietary intake of hydroxycinnamic acids, the most consumed polyphenolic family in this population.

**Conclusions:**

A bicarbonate-based Folin-Ciocalteu micromethod allowed the accurate determination of polyphenols in HM, which might be useful for translational research settings and HM banks.

## RESUMO

**Objetivo:**

Desenvolver um método rápido para analisar polifenóis, que são antioxidantes potencialmente ativos contra o estresse oxidativo neonatal, em pequenos volumes de leite humano (LH).

**Métodos:**

Foram comparadas extrações ácidas e alcalinas usando dois corantes: Folin-Ciocalteu e Fast Blue BB. Foram avaliadas variáveis como linearidade, sensibilidade, percentagem de recuperação, teor de polifenóis, precisão e estabilidade em 14 amostras de LH, comparadas usando o teste de Kruskal-Wallis H (p<0,05). A melhor técnica foi aplicada a 284 amostras de LH para determinar seu teor polifenólico e sua associação com a dieta materna por regressão linear multifatorial.

**Resultados:**

A extração ácida recuperou com sucesso o padrão de referência do ácido gálico, enquanto a extração alcalina o superestimou. As curvas de calibração para todos os métodos foram lineares (R^2^>0,96) até os 500 mg/L. Todos os métodos testados baseados em Folin-Ciocalteu com bicarbonato foram estáveis e repetíveis, enquanto as variantes baseadas em Fast Blue BB não. Os polifenóis do HM (média=94,68 mg/L) correlacionaram-se positivamente com a ingestão dietética de ácidos hidroxicinâmicos, a família de polifenóis mais consumida nesta população.

**Conclusões:**

Um micrométodo baseado em bicarbonato de Folin-Ciocalteu permitiu a determinação precisa de polifenóis no HM, o que pode ser útil para configurações de pesquisa translacional e bancos de HM.

## INTRODUCTION

Oxidative stress is the principal mechanism underlying typical prematurity-related diseases such as bronchopulmonary dysplasia, retinopathy, necrotizing enterocolitis, intraventricular haemorrhage, kidney damage, eryptosis, respiratory distress syndrome, and patent ductus arteriosus.^1^ Additionally, preterm infants are the principal users of human milk (HM) banks associated with neonatal care services. Consequently, the antioxidant content of HM must be known to define neonatal requirements, and its dosage is necessary to prevent these diseases and their sequelae by personalising nutrition.^2^ Thus, practical analytical solutions are needed to determine antioxidant compounds in HM.

The scientific community has shown interest in the functional compounds present in HM, including polyphenols, which are phytochemicals that are not synthesised nor stored by humans. Therefore, they are incorporated only by the intake of plant-based foods.^3^ Their bioavailability in HM subsequently depends on the maternal diet and other health conditions.^4^ These compounds are powerful antioxidants that reduce oxidative stress in premature newborns, who are highly vulnerable to reactive oxygen species given their deficient antioxidant defences.^5^ Phytochemical intake through breastfeeding may prevent these disorders by enhancing free radical scavenging,^6^ and polyphenol quantification is therefore encouraged to identify an adequate antioxidant supply.

Little is known about the total polyphenol content in HM,^7^ because there is a lack of validated analytical techniques for this fluid. Therefore, this study aimed to develop and apply an appropriate, inexpensive, and rapid colorimetric micromethod for the extraction and quantification of polyphenols in HM. Additionally, the developed micromethod will be validated using maternal polyphenolic dietary intake as a reference. This will make it possible to identify donated HM in banks with significant antioxidant potential. Thus, meaningful results might be obtained by applying translational research on HM, which produces direct benefits by efficiently translating basic scientific discoveries into practical applications.^8^ This is particularly important for low- to middle-income regions, such as Latin American countries, because the current analytical techniques are difficult to implement. They require many expensive reactants, high sample volume with complex processing, specialised human resources and equipment.^9^

## METHOD

An experimental and cross-sectional study was conducted. The Human Milk Bank in the “Ministro Dr. Ramon Carrillo” of the Maternal-Neonatal Hospital (Cordoba, Argentina) provided 14 HM samples from July to August 2022. Samples were stored immediately after extraction in an ultra-low freezer at -80°C (Model 706, Thermo Scientific, USA) to avoid deterioration. Additionally, an analytical scale with a readability of 0.0001 g (Nimbus, Adam Equipment, USA), a vortex mixer with a speed range of 0–3,000 rpm (Vortexer, Heathrow Scientific, USA), microwave oven (WMD20GS, Whirlpool, Argentina), a refrigerated centrifuge (EPP-24, Zelian, Argentina), and an incubator (LTE Scientific Ltd., UK) were used for sample processing. Ultrapure water was obtained with a water purification system (Barnstead Easypure II 7031; Thermo Scientific, USA). For sample analysis, a GloMax Multidetection System 96-well microplate reader was employed (Promega, USA) with a 450–750 spectral range, 0–4.0 OD photometric measuring range, and <0.2% precision.

Regarding the employed reagents, gallic acid was used as the reference standard (C_7_H_6_O_5_, 99.6% purity; Anedra, Argentina). For polyphenol extraction methods, methanol (CH_3_OH, 99.8% purity, Cicarelli, Argentina), trichloroacetic acid (C_2_HCl_3_O_2_, 99.23% purity, Parafarm, Argentina), and sodium hydroxide (NaOH, 97% purity, Cicarelli, Argentina) were used. Colorimetric assays to determine the polyphenol content of HM required Folin-Ciocalteu (FC) reagent (2N, Tetrahedron, Argentina), Fast Blue BB (FBBB) salt (C_17_H_20_N_2_O_3_, 95% purity, Sigma-Aldrich, USA), sodium bicarbonate (NaHCO₃, 99.7% purity, Cicarelli, Argentina), and sodium carbonate (Na₂CO₃, 99.5% purity, Merck, Germany).

**Figure 1:**
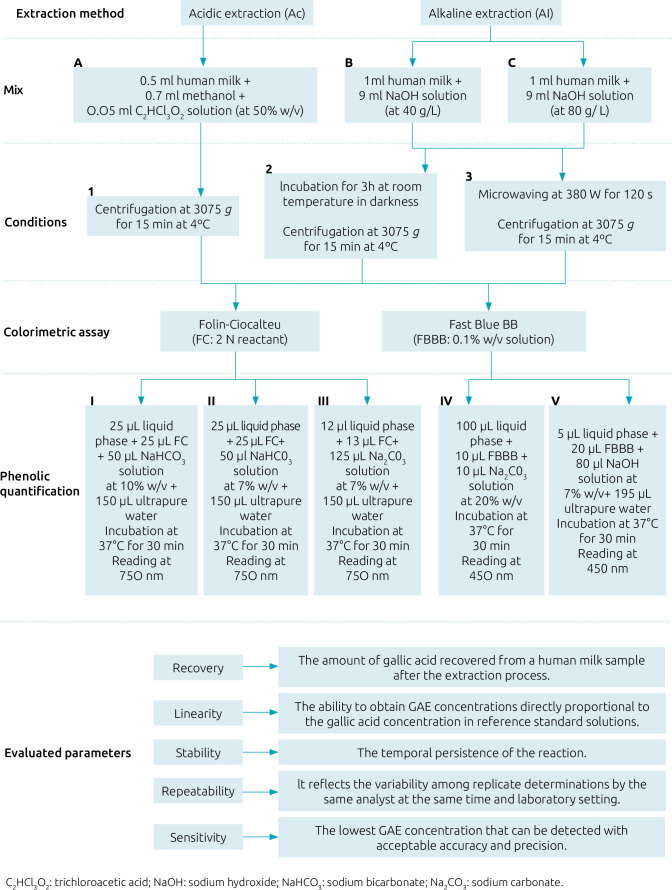
Algorithm for technical comparison to study polyphenols in human milk.

Ultrapure water was the solvent for all the solutions unless otherwise specified. Two types of methods were compared for extracting HM polyphenols: acidic and alkaline methods (Figure 1). For acidic extraction, each sample (0.5 mL) was mixed with 0.7 mL of methanol and 0.05 mL of a trichloroacetic acid solution (50% w/v) (A).^8^ For alkaline extraction, traditional and microwave-assisted methods were used.^10,11^ For both alkaline extractions, 1 mL of HM was mixed with 9 mL of either a 40 g/L (B) or 80 g/L (C) solution of NaOH. For the traditional extraction method, solutions were incubated at room temperature for three hours in the dark (2), whereas for the microwave-assisted extraction they were microwaved at 380 W for two minutes (3). Finally, all solutions were centrifuged at 3075 × g for 15 min at 4°C (1, 2, 3) to obtain the liquid upper phase (acidic samples) or lower phase (alkaline samples) (Figure 1).

After extraction, two reactants were used to determine polyphenols in all liquid-phase samples: FC and FBBB.^12^ Three variants of the FC assay were evaluated using different solutions for pH alkalinisation: sodium bicarbonate at 10% (I) or 7% w/v (II), or sodium carbonate at 7% w/v (III). Then, 25 μL of the previously-obtained liquid phases were mixed with either 25 μL of 2N FC solution and 50 μL of one of the sodium bicarbonate solutions, or 13 μL FC and 125 μL of the sodium carbonate solution.^7,13^ Finally, 150 μL of ultrapure water were added to each solution and incubated at 37°C for 30 min and read at 750 nm^14^ (Figure 1).

Two variants of FBBB were compared: for the first, 100 μL of each liquid phase were mixed with 10 μL of a 0.1% w/v FBBB solution and 10 μL of sodium carbonate at 20% w/v (IV);^13^ for the second, 5 μL of the liquid phase were mixed with 20 μL of the same FBBB solution, 80 μL of 7% w/v NaOH, and 195 μL of ultrapure water (V).^14,15^ Then, all samples were at 37°C for 30 min and reading at 450 nm.

For each method, total polyphenol content was expressed as mean mg/L of gallic acid equivalents (GAE) from triplicate analyses (Figure 1).

Validation was performed according to current guidelines,^16,17^ with gallic acid as the reference standard. All procedures were performed in triplicate for the three HM samples unless otherwise specified. All statistical analyses were performed on STATA 15.

For analytical linearity and sensitivity, three reference standard solutions of 4000 mg/L gallic acid were prepared using the three solutions used for the polyphenolic extraction (A, B, C) (Figure 1). Subsequently, ten dilutions were obtained from each solution for the calibration curve, which ranged from 0 to 4000 mg/L. The linear relationship between gallic acid concentration and absorbance at 750 nm (for FC) or 450 nm (for FBBB) was expressed using linear regression coefficients and R^2^ values. The limit of blank (LoB) and limit of detection (LoD) were verified for each method. For LoB, the FC and FBBB methods were performed in 15 replicates of a blank sample to calculate their mean absorbance and standard deviation (SD). The LoB was calculated as mean±1645 × SD. The LoD is the lowest GAE concentration that is likely to be reliably distinguished from the LoB, at which detection is possible. It was determined as LoB + 1.645 × SD of the lowest GAE concentration sample.^18^

For verifying polyphenol recovery from HM, gallic acid was added as a standard for each extraction solution at a final concentration of 500 mg/L. Recovery percentages were calculated as follows: (([GA] sample with standard − [GA] sample without standard)/[GA] standard) × 100, expressed as mean±SD for each polyphenol extraction method.^19^ Means and SD were reported. Differences between the methods were explored using the Kruskal-Wallis H test at a significance level of p<0.05. This statistical treatment was also applied during the polyphenol quantification in the HM samples.

Repeatability was calculated by processing triplicate aliquots of the 14 HM samples for each proposed quantification technique, after selecting the best extraction method. Then, coefficients of variation were calculated. All samples were prepared by the same analyst in the same laboratory setting on the same day. Additionally, stability was assessed by determining the absorbance of each sample at 30, 45, 60, 75, 90, and 105 minutes and calculating the percentage of stability with respect to 30 minutes.

Technology Readiness Level 7 requires innovations to be demonstrated on a larger scale in a real-life setting.^20^ Thus, the selected technique after the validation process was applied to 284 HM samples collected from 2013 to 2020 from Cordoba (Argentina) and stored in a bank. Their donors provided the epidemiological information necessary to perform the subsequent analyses.

First, multivariate imputation with chained equations was used to impute 31 missing values for milk polyphenols (percentage missing=10.92). Missing at random was assumed as the missingness mechanism. To improve imputation accuracy, several auxiliary variables were used for being theoretically related to the to-be-imputed variables: age, postpartum time, parity, exclusive breastfeeding practice, nutritional status (body mass index, body fat percentage, and physical activity level), dietary indicators (dietary pattern indices, energy intake, polyphenol intake, protein/carbohydrate ratio, and fat quality index),^21^ and milk biomarkers (triacylglycerols, cholesterol, glucose, proteins, lipoperoxides, hydroperoxides, and superoxide anions). Twenty imputed datasets were created, and the results were combined to account for uncertainty in the imputed data.

The variable distributions did not differ substantially between the participants with missing and imputed data. The association between HM polyphenol content and dietary polyphenol intake was studied using linear regression adjusted for postpartum time, exclusive breastfeeding occurrence, body fat percentage, metabolic equivalents of task, dietary adherence scoring, protein/carbohydrate ratio, fat quality index, total energy value, and minor intake of other polyphenols. The maternal polyphenol consumption was assessed by the HJ-biplot technique, grouping women according to the intake level of major dietary polyphenols into three clusters:^22^

- C1: women with low intake of any compound, with total polyphenol mean (SD) in diet being 538.59 (241.35) mg/d (range: 0–1206.48).
- C2: women with high intake of diverse compounds (hesperetin, caffeic acid, lariciresinol, and ferulic acid), with total polyphenol mean (SD) in diet being 1436.24 (390.27) mg/d (range: 825.78–2884.53).
- C3: women with high intake of hydroxycinnamic acids, with total polyphenol mean (SD) in diet being 1405.25 (533.60) mg/d (range: 585.03–2923.86).

The results were expressed as coefficients with standard error (SE) and p-values. In addition, given the inclusion of multiple covariables for linear regression, the achieved power (1-β error probability) was 0.9999895 computed *post hoc* using a random model for exact distribution (two tails, H1 p²=0.2165053, H0 p²=0, observed R^2^=0.25, α error probability=0.05, sample size=284, number of predictors=14) using G*Power 3.1.9.7.

Written informed consent was obtained from all participants. The Research Ethics Committee of the National Clinics Hospital (National University of Cordoba) approved this study in accordance with the Declaration of Helsinki and current legislation (codes: RENIS-IS000548/IS001262/IS002045; REPIS145/2654/5554).

## RESULTS

All methods’ R^2^ were >0.96. Also, determinations performed after acidic extraction exhibited mean values of total polyphenols within the acceptable range of linearity (Table 1). Calibration curves showed a linear response to the gallic acid concentration, with a lower limit of 7.8 mg/L and an upper limit ranging from 500 to 4000 mg/L. Regarding sensitivity, method A-I showed the lowest estimated LoB and LoD (5.84 and 8.08 mg/L, respectively) (Table 1).

**Table 1:** Linearity and sensitivity of different methods to determine polyphenols using a standard curve of gallic acid.

Method	Linear equation	R^2^	Upper limit (mg/L)	Limit of blank (mg/L)	Limit of detection (mg/L)
Folin-Ciocalteu					
A–I	y=0.1062x+0.0073	0.9874	500	5.84	8.08
A–II	y=0.1484x-0.0109	0.9993	500	7.67	28.57
A–III	y=0.3225x-0.0321	0.9982	2000	9.71	11.77
B–I	y=0.9289x-0.0997	0.9645	4000	38.50	59.43
B–II	y=0.8708x-0.1074	0.9864	4000	78.37	112.83
B–III	y=2.0941x-0.1895	0.9833	4000	71.17	79.18
C–I	y=0.5816x-0.0143	0.9935	2000	15.18	21.90
C–II	y=0.5975x-0.0218	0.9860	2000	36.93	69.84
C–III	y=1.3593x-0.1032	0.9847	4000	62.49	75.14
Fast Blue BB					
A–IV	y=0.9339x-0.1985	0.9724	4000	8.46	16.66
A–V	y=8.8109x-0.8807	0.9856	4000	55.77	88.41
B–IV	y=1.1998x-0.1815	0.9957	4000	62.43	105.78
B–V	y=8.4058x-0.7859	0.9849	4000	255.87	287.57
C–IV	y=0.8398x-0.1217	0.9920	4000	74.59	101.72
C–V	y=5.9744x-0.5567	0.9907	4000	140.37	188.09

Acidic extraction followed by centrifugation recovered the total amount of the gallic acid standard. Conversely, the other procedures reported values greater than expected, indicating that they extracted other reactive compounds (Table 2). Data dispersion was derived from analysing together FC and FBBB outcomes per extraction method, which can vary according to their technical features.

**Table 2:** Polyphenol recovery (%) using gallic acid as standard in human milk according to extraction methods.

Method	Mean	SD	Grouping*		
A–1	115.85	62.52	A		
C–3	596.84	341.83		B	
C–2	587.08	292.05		B	
B–3	843.27	440.63		B	C
B–2	2233.07	1774.71			C

*The same letter indicates no significant difference in the Kruskal-Wallis test for p<0.05.

There were significant differences in the mean total polyphenol contents among the studied methods. The lowest concentration corresponded to method A-1-II and was 58.75 (33.17) mg/L, whereas the highest concentration was 7777.42 (3235.98) mg/L for method B-3-III. Assays using acidic extraction did not differ significantly (except for A-1-II and A-1-V), whereas alkaline extraction exhibited significant variability (Table 3).

**Table 3:** Total polyphenol content (mg/L) found by different methods in human milk.

Method	Mean	SD	Grouping*									
A–1–II	58.75	33.17	A									
A–1–I	70.54	17.40	A									
A–1–III	205.08	423.04	A	B								
A–1–IV	311.77	425.15	A	B								
C–3–IV	730.40	205.00	A	B	C							
C–2–IV	1246.41	506.50	A	B	C	D						
A–1–V	1612.23	1692.27		B	C	D	E					
C–3–V	2239.55	1121.29			C	D	E	F				
B–2–II	2176.99	837.86			C	D	E	F				
C–2–II	2301.46	183.68			C	D	E	F				
C–2–I	2417.33	491.06				D	E	F				
C–3–I	2690.68	942.71				D	E	F				
C–2–V	2790.52	1323.99				D	E	F				
C–3–II	2747.59	998.08				D	E	F				
B–2–I	2861.03	857.91					E	F	G			
B–2–IV	3120.18	1410.01					E	F	G	H		
B–3–II	3323.54	1481.97					E	F	G	H	I	
B–3–IV	3600.10	794.34						F	G	H	I	J
B–3–I	4092.34	1856.35						F	G	H	I	J
C–3–III	7161.56	3465.90							G	H	I	J
B–2–III	5762.43	1281.94								H	I	J
B–2–V	7243.42	4186.90									I	J
B–3–V	6449.54	1916.71									I	J
B–3–III	7777.42	3235.98									I	J
C–2–III	7493.83	941.33										J

*The same letter indicates no significant difference in the Kruskal-Wallis test for p<0.05.

Fourteen methods reported means within the linearity range (A-1-II, A-1-I, A-1-III, A-1-IV, C-3-IV, C-2-IV, A-1-V, B-2-II, C-3-V, C-2-V, B-2-I, B-2-IV, B-3-II, and B-3-IV). Among these, method A was the most sensitive (Table 1), with A-1 recovering polyphenols appropriately (Table 2).

**Figure 2:**
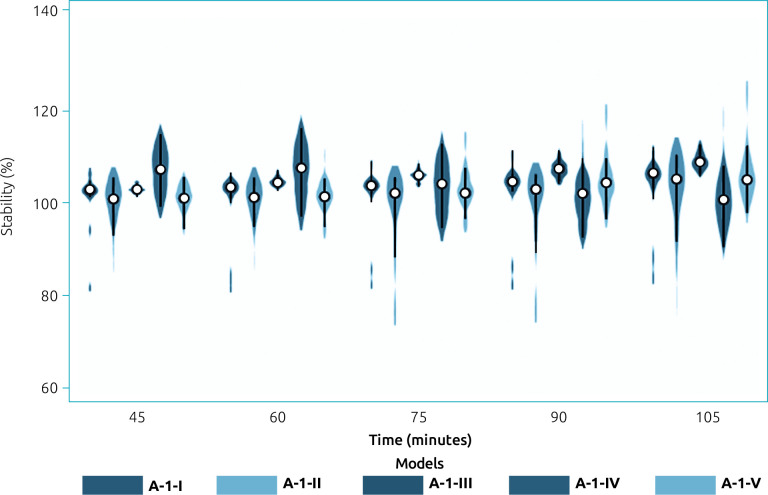
Percentage of stability of the A-1 methods for polyphenol quantification in human milk, with respect to 30 minutes. A-1 methods consist of acidic extraction accompanied by centrifugation at 3075 g for 15 min at 4°C. Different conditions were tested for colorimetry, which was based on Folin-Ciocalteu reagents (I, II, and III) and Fast Blue BB (IV and V).

Given the above-mentioned results, the following steps used only A-1 methods. Regarding the stability of the reactions, all five methods remained stable during the assay, as shown in Figure 2. These methods exhibited the following coefficients of variation at a reaction time of 30 min: A-1-I=0.10, A-1-II=0.14, A-1-III=0.08, A-1-IV=0.12, and A-1-V=0.18, with the first and third methods being the most repeatable.

Using A-1-I, considered as the best method, polyphenols averaged 94.68 (26.26) mg per litre of HM in a sample of 284 women, with a range of 45.21–174.99 mg/L. This value was not significantly different from the previous value reported in Table 3, supporting the external validity of the results. An increase in milk polyphenol content was observed due to the intake of hydroxycinnamic acids (C3: coefficient=9.92, SE=4.99, p<0.05) compared to the low intake group (C1), whereas the other dietary polyphenols did not contribute to increasing milk levels (C2: coefficient=1.34, SE=5.84, p>0.05).

## DISCUSSION

This study aimed to develop a rapid method for determining total polyphenols in small volumes of HM using assays involving either the FC or FBBB reactants. In this sense, all assays were highly linear, which indicated the reactivity with polyphenols under the physicochemical conditions used.

The acidic extraction had a better performance, obtaining linearly-ranged mean values. In this sense, trichloroacetic acid decreases the pH and precipitates proteins. This contributes to avoiding overestimation,^23^ given that proteins also react in colorimetric assays. In contrast, alkaline extraction greatly hydrolyses proteins. This extraction method has been successfully applied in plant tissues,^24^ but not in HM. Nonetheless, the composition of HM does not make it a good candidate for this extraction because its significantly complex matrix is an interferer.^1^ In this sense, the alkaline extractions assayed in this study greatly overestimated polyphenols (with means ranging from 587.08 to 2,233.07%). Conversely, a low pH increases the levels of the non-dissociated forms of phenolic acids, such as gallic acid, which favours their solubility in methanol.^25^ This organic solvent enhances protein denaturation,^26^ leading to a homogeneous liquid phase better suited for polyphenol quantification.

The A-1 methods achieved a complete gallic acid recovery. Among those, method A-I-1 (which used a saturated sodium bicarbonate solution) was the most sensitive. FC forms a blue phosphotungstic-phosphomolybdenum chromophore, with maximum absorption depending on pH and phenolic content.^27^ This is best achieved with the A-I-1 method, which uses a higher bicarbonate concentration in a lower final reaction volume. Thus, a more diluted solution (e.g., A-1-II) is insufficient to provide the optimal chemical conditions for the reaction between the reactant and the standard solution. Conversely, diprotic sodium carbonate (e.g., A-1-III) has different properties from monoprotic sodium bicarbonate. In this sense, bicarbonate is a less caustic base with good electrochemical profile, which is beneficial given that the rapid FC decomposition impairs quantification.^27^

Although FBBB-based methods are preferred for a variety of tissues,^13^ there are no reports on the use of this reactant in HM. To date, the only colorimetric assay performed on milk from several mammalian species (including HM) uses the FC reactant.^7^ In this study, FBBB presented lower technical quality indicators (such as sensitivity and recovery) than FC.

Eleven methods (B-2-III, B-2-V, B-3-I, B-3-III, B-3-V, C-2-I, C-2-II, C-2-III, C-3-I, C-3-II, and C-3-III) resulted in milk polyphenols out of the linear range, with mean values greatly exceeding those of previous reports.^7,8^ Therefore, these results were deemed implausible. Among the other assays, A-1-I and A-1-II were the least variable, therefore being more reliable. These FC-based methods were both linear and sensitive. Consequently, 58.75 and 70.54 mg/L of polyphenols were plausible outcomes. These concentrations are close to those established in the scientific literature,^6^ although other authors report different values.^28^

Regarding precision, A-1-I and A-1-III showed better repeatability after 30 minutes of reaction. Consequently, the differences in repeatability were responsible for the dispersion observed during the stability assessment. All reactions were stable for up to 105 min. Concerning this, A-1-I was consistently a more precise method in the current study, which supports its repeatability. The discordance between intra-individual coefficients of variation and inter-individual standard deviations found with A-1-III indicated that this method is not reliable, despite having previously been shown to be linear, sensitive, and repeatable. In other words, although it was repeatable for each sample, it was not consistent among different samples, suggesting the quantification of other compounds.

Polyphenols are phytochemicals provided by diet.^29^ Consequently, the determination of polyphenols in HM requires establishing a diet-milk relationship to be valid. In this study, milk polyphenols were positively associated with maternal intake of hydroxycinnamic acids. These compounds are the most consumed in this population,^22,30^ which supports their contribution to polyphenols in HM. Other polyphenolic families are less ingested (e.g., flavonoids),^22^ and consequently their contribution to HM was not significant. Thus, milk content enhancing requires a higher intake, as is the case of other populations.^3^

This study has some limitations. High-performance liquid chromatography could identify each individual compound contributing to the total polyphenolic content determined by spectrophotometry.

Additionally, examining inter-operator variability would provide further insights into the validity of the method. Thus, it is recommended that future studies address these aspects. Nonetheless, our results are supported by a rigorous and comprehensive validation process.

Polyphenols promote breastfeeding benefits as they are antioxidant and chemopreventive agents that protect maternal and infant health. Given this, A-1-I could be routinely used in neonatal units and HM banks to estimate polyphenol content in HM, since this method proved to be the most accurate measurement of polyphenols, requiring only a minimal amount of sample without compromising the provision of newborn HM. It demonstrated superior linearity, sensitivity, efficiency, repeatability, and stability compared to other assays. This colorimetric assay, based on the FC reactant, is easy, fast, and affordable, requiring only readily available equipment. Furthermore, its validity is supported by the responsiveness of its outcomes to the corresponding dietary supply in a population-based study.

## Data Availability

The database that originated the article is available with the corresponding author.
